# THE CURRENT SITUATION AND INFLUENTIAL FACTORS OF FRAILTY IN ELDERLY PATIENTS WITH DIABETES IN CHINA

**DOI:** 10.1093/geroni/igz038.3085

**Published:** 2019-11-08

**Authors:** Fumin Dai, Wenwen Jia, Mengdie Jiang

**Affiliations:** 1 Henan Provincial People’s Hospital, Zhengzhou, Henan province, China; 2 Henan University, Zhengzhou, Henan, China

## Abstract

Patient with diabetes may increase the incidence of frailty. Frailty cause chronic inflammation and insulin resistance. The purpose of this study is to investigate the current situation of frailty and its influencing factors in elderly patients with diabetes. Totally 300 elderly patients with diabetes were selected from a tertiary hospital in Zhengzhou of China via convenience sampling method，and were investigated by self-designed general information questionnaire，TFI (Tilburg Frailty Indicator) and SDSCA(Scale of Diabetes Self-care Activities). Totally 296 valid questionnaires were collected. A total of 137 elderly patients with diabetes suffered from frailty and the prevalence was 46.3%. The mean score of total frailty was (5.26±2.87) and the scores for each dimension were as follows: physical frailty (2.79±2.08), psychological frailty (1.40±0.94) and social frailty (1.07±0.75). Multiple linear regression showed that comorbidity, self-management behavior, glycosylated hemoglobin, educational degree, polypharmacy and smoking were the major influential factors (P<0.05). The prevalence of frailty in elderly patients with diabetes was at a high level, containing different degrees of physical, psychological and social frailty. Medical staff should attach great importance to the assessment of frailty among elderly patients with diabetes, take targeted and holistic interventions timely to prevent or delay the development of frailty.

